# Regulation of the innate immune response in rheumatoid arthritis

**DOI:** 10.3389/fimmu.2025.1545625

**Published:** 2025-11-03

**Authors:** Aikeremujiang Muheremu, Shalayiding Aierxiding, Jian Gao

**Affiliations:** ^1^ Department of Orthopedics, Sixth Affiliated Hospital of Xinjiang Medical University, Urumqi, Xinjiang, China; ^2^ Key Laboratory of Orthopaedic Regenerative Medicine, Sixth Affiliated Hospital of Xinjiang Medical University, Urumqi, Xinjiang, China

**Keywords:** rheumatoid arthritis, innate immune response, innate immune system, adaptive immune system, regulation

## Abstract

Rheumatoid arthritis (RA) is a common inflammatory joint disorder characterized by progressive joint destruction, leading to painful deformity and limited mobility, for which there is currently no effective cure. While existing treatments offer symptom relief, a significant unmet need persists due to inadequate disease control in many patients. Global epidemiological studies estimate that the current incidence of RA ranges from 20 to 50 cases per 100,000 people annually, with a prevalence of 0.5% to 1.0%. The disease disproportionately affects women (2-3 times more than men) and peaks in older age groups (65-69 years). This ongoing disease burden highlights the importance of understanding its underlying mechanisms. RA is a complex autoimmune condition with a multifactorial etiology involving genetic susceptibility, environmental factors, and dysregulated immune responses characterized by autoantibody production, chronic inflammation of the synovium, and progressive joint damage. Its pathogenic process involves activating various immune cells, which significantly contribute to disease development. Extensive experimental research and clinical trials have demonstrated the different roles of the Innate Immune System (IIS) in RA. However, the scientific community remains divided on defining the composition and functions of the IIS during the onset and progression of RA. Therefore, it is essential to further investigate the role of the IIS and its relationship with the Adaptive Immune System (AIS) in RA treatment. This review covers the regulatory effects and biological functions of the Innate Immune Response (IIR), explores the underlying mechanisms of RA, and offers insights into potential biomarkers and therapeutic strategies, enhancing current understanding of the IIR for future research.

## 1 Introduction

**Figure 1:**
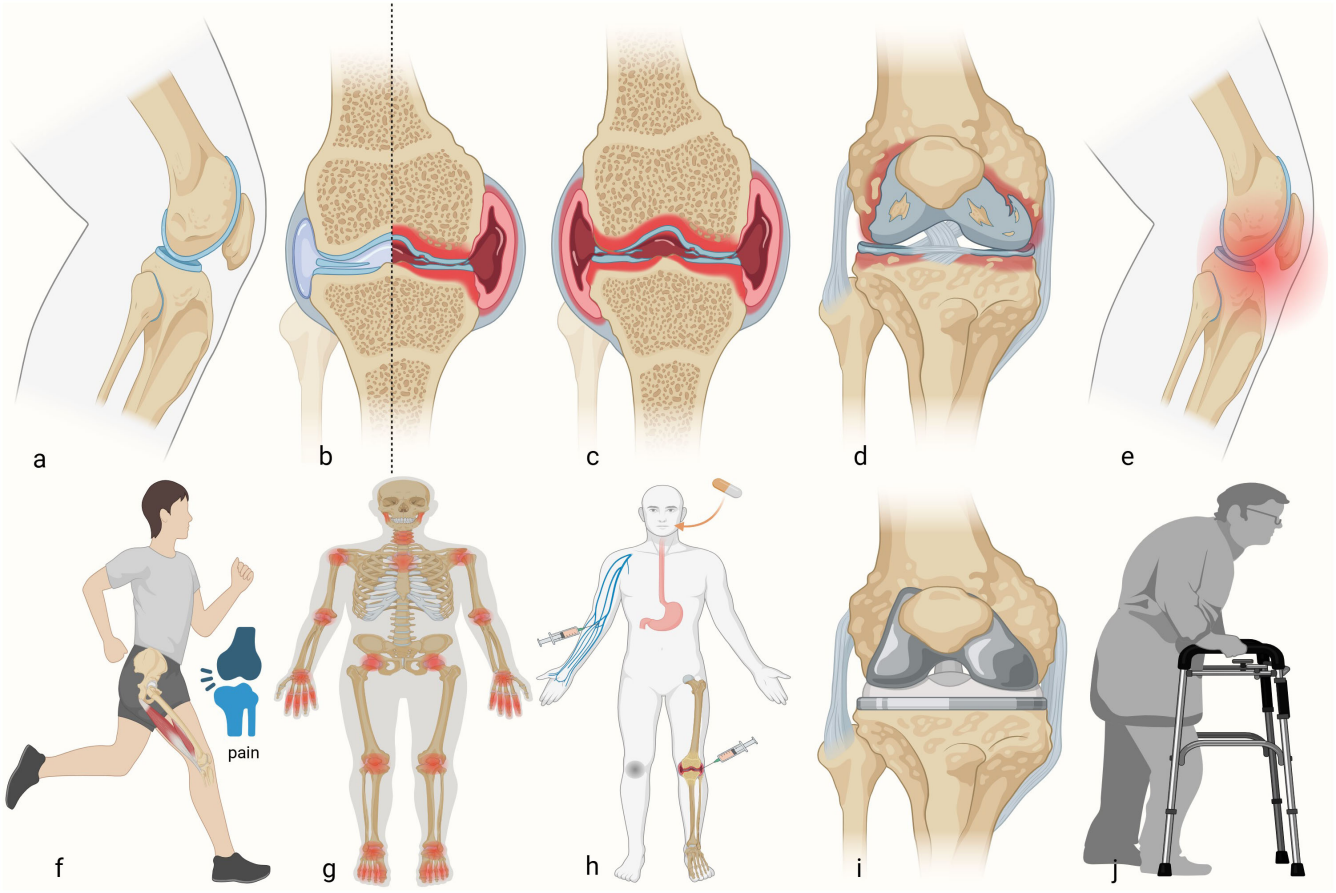
Developmental process of rheumatoid arthritis. **(a)** Healthy knee. **(b)** Schematic of the RA damage to half of the knee. **(c)** RA developed into the whole knee. **(d)** Bone and cartilage injuries. **(e)** The occurrence of knee pain. **(f)** Impairment of motor function. **(g)** RA accumulation joints of the whole body. **(h)** Different kinds of treatment for patients with RA in the early stage. **(i)** Total knee arthroplasty in advanced patients. **(j)** Postoperative rehabilitation training.

RA is a chronic, autoimmune, systemic disease characterized by persistent joint inflammation, leading to pain, swelling, stiffness, and functional impairment (1, 2). These clinical manifestations stem from complex immunopathological processes that remain incompletely understood. Globally, the incidence of RA is approximately 40 per 100,000 individuals per year, with a prevalence of around 0.5%-1%, and the average age of onset is between 40 and 60 years, with a sex ratio of 1:2 to 1:3 (male: female) (3, 4). Recent studies provide more granular epidemiological insights: According to Kim H. et al., seropositive RA is one of the most prevalent autoimmune rheumatic diseases (AIRDs) in Korea, with 96,330 cases, which is 188.5 per 100,000 population (5), and a recent study shows that the prevalence of RA in the USA ranges between 0.5-1.0% (6). The causes and development of RA have been extensively explored, yet a complete understanding of RA continues to elude us. Given the complex interplay of genetic and environmental factors in RA pathogenesis, increasing attention has focused on immune system dysregulation as a key driver of disease progression. With the deepening of research, it has become increasingly recognized that the IIS plays an important regulatory role in the pathogenesis of RA. (**Figure 1**).

**Figure 2:**
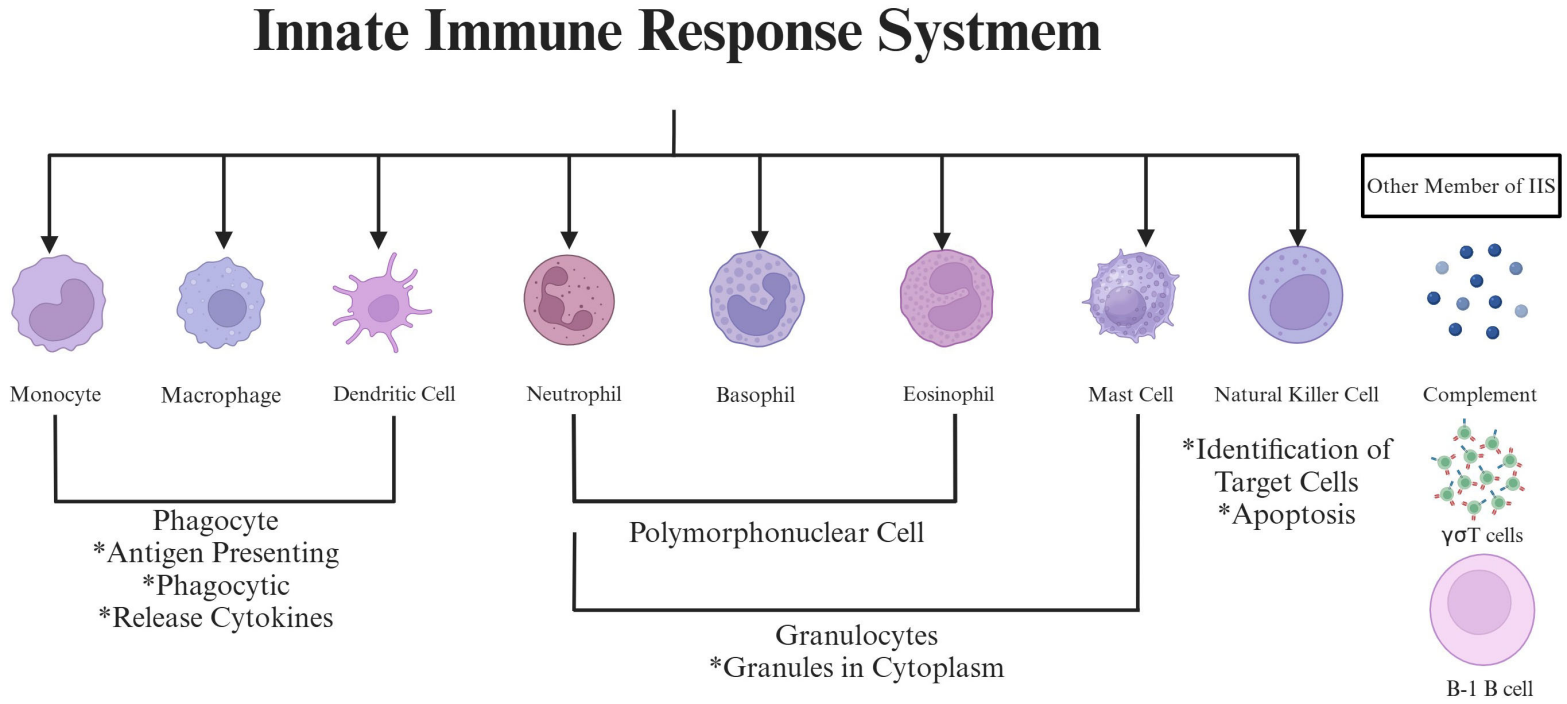
The schematic picture of the innate immune response system. This picture highlights key cellular elements (monocytes/macrophages, dendritic cells, neutrophils, basophils, eosinophils, mast cells, natural killer cells, and B-1 B cells) and complement system components involved in innate immunity. Functional features like phagocytosis, antigen presentation, cytokine release, granulocyte-mediated cytotoxicity, and target cell recognition play a role in synovial inflammation by working together in RA progression.

To fully appreciate the IIS’s involvement in RA, it is essential to first understand the immune system’s broader structure and function. The immune system is one of the most complex biological systems known to humans, second only in importance and complexity to the nervous system (7). The immune system is a delicate network composed of cells and effector molecules, designed to defend against invaders while protecting the integrity of “self” and facilitating recovery (8). This network operates through two interconnected branches: It includes IIS and the AIS, which must collaborate effectively to guarantee the optimal operation of the body (9). Of these two branches, the IIS is particularly relevant to RA pathogenesis due to its role as the body’s first responder to inflammation and tissue damage (10). IIS comprises numerous components, including immune cells, soluble recognition molecules, and the complement system (11). Within this framework, key cellular constituents of IIS encompass phagocytes (such as macrophages), antigen-presenting cells (such as dendritic cells), and cytotoxic cells (such as natural killer cells) (12). Some literature also includes NK T cells, neutrophils, basophils, eosinophils, B-1 cells, and γσT cells within the IIS framework (13). (**Figure 2**).

The transition from protective immunity to pathological inflammation in RA involves specific IIS activation pathways. IIS activates various immune cells and the complement system through pattern recognition receptors (PRRs), pathogen-associated molecular patterns (PAMPs), and damage-associated molecular patterns (DAMPs), thereby generating and promoting inflammatory responses (13). In the context of RA, the abnormal activation of the IIS is a key element in its pathogenesis. In the synovial joint, innate immune cells such as macrophages, dendritic cells, neutrophils, natural killer cells, and mast cells are highly active in RA (14). These activated cells drive disease progression through multiple mechanisms: They can promote the development of synovial joint inflammation by releasing inflammatory cytokines, chemokines, activating the complement system, and performing phagocytosis or antigen presentation functions (15). Therefore, modulating the IIS response in RA, particularly controlling its activation and the mediated inflammatory response, has become an important strategy in treating RA (16). Recent advances have shed new light on regulatory mechanisms targeting the IIR. While challenges remain, to effectively modulate the immune response in RA, it is necessary to overcome the sustained activation of IIS and address the activation of AIS associated with autoantibody production, which continuously fuels the chronic inflammatory process in the synovium.

The therapeutic targeting of immune modulation has evolved significantly over time. The concept of overcoming RA through the supplementation of immune-modulating mediators is not novel; for instance, in the late 1990s, IL-11 was considered an important regulatory cytokine in RA (17). This early approach represented one of the first attempts to rebalance the immune system rather than simply suppress inflammation. Almost simultaneously, it was proposed by scholars that interferon β (IFNβ) could serve as an anti-inflammatory mediator in RA (18). Initial optimism was supported by findings that showed IFNβ alleviated arthritis in mice and demonstrated clinical relief of symptoms and signs in preliminary studies on RA patients, yet later studies on IFNβ in RA yielded discouraging results (19). This pattern of initial promise followed by disappointment characterized subsequent hypotheses regarding immune-modulating mediators for treating RA, as these approaches yielded either negative or mixed results, leading to temporary abandonment of cytokine modulation strategies (20).

However, the field has experienced a renaissance with discoveries reinvigorating the idea that specific anti-inflammatory cytokines may inhibit RA disease progression, and even promote joint cartilage repair, by modulating IIS or AIS. Modern approaches now focus on regulating macrophage polarization direction, complement system activation, antigen-presenting cell function, neutrophil activity, and effectively inhibiting B-cell or T-cell activation through more targeted mechanisms. In light of these developments, IIS plays a crucial role in the pathogenesis of RA. Research into its regulatory mechanisms not only aids in deepening our understanding of the pathological processes of RA but also offers potential for developing new therapeutic strategies. Moving forward, future research is needed to elucidate the targets through which innate immune cells regulate inflammatory responses in RA and how these targets can be effectively intervened upon to control or reverse disease progression. This comprehensive review examines the mechanisms by which key members of the IIS regulate immune responses in RA, bridging historical perspectives with contemporary therapeutic opportunities.

## 2 Regulation of macrophages in RA

Macrophages serve as central orchestrators of RA pathogenesis through their dual roles in inflammation and tissue homeostasis. Recent research indicates that various innate immune cells within synovial tissue, particularly monocyte-derived macrophages, contribute to the inflammatory response and play a pivotal role in activating AIRDs (21). As the first responders in RA joints, macrophages represent the earliest and most abundant immune cells in affected synovium (22), where they drive pathology through multiple mechanisms: production of pro-inflammatory cytokines, including tumor necrosis factor-α (TNF-α), IL-1β, and IL-6 (23), chemokines such as Chemokine (C-C motif) ligand 2 (CCL2), and tissue-destructive metalloproteinases (MMP-3, MMP-12) (24).

Macrophage polarization exists along a spectrum with two well-characterized extremes: classically activated M1 macrophages, which promote joint erosion through secretion of pro-inflammatory cytokines (TNF-α, IL-1) (25), and alternatively activated M2 macrophages that secrete anti-inflammatory mediators (IL-10, TGF-β) to facilitate tissue repair and remodeling (26). This polarization continuum includes intermediate states marked by distinct surface marker profiles and functional outputs, including nitric oxide and cytokine production (27). These factors collectively delineate the morphological and functional states of macrophages. RA is frequently linked to inappropriate interactions between macrophages and T cells, where the activation of M1 or M2 macrophages can significantly influence the emergence of Th1 or Th2 responses in helper T cells (28). The macrophage-T cell crosstalk in RA creates a self-perpetuating inflammatory cycle. M1 macrophages preferentially drive Th1 responses through TLR/IFN signaling (29), generating a cascade of destructive mediators including TNFα, IL-12, IL-18, IFNγ, and matrix metalloproteinases that promote osteoclastogenesis and joint destruction (30). Conversely, M2 macrophages induce Th2 responses characterized by IL-4, IL-10, IL-13, and TGF-β secretion, which correlate with clinical remission (31). This immunological imbalance - with predominant M1/Th1 activation in RA - highlights the pathogenic consequences of disrupted macrophage polarization.

The functional dichotomy between macrophage subsets is central to RA pathogenesis. M1 macrophages are recognized as pro-inflammatory cells, exhibiting elevated expression of major histocompatibility complex (MHC) class II, CD80, CD86, CD38, and TLR4, which contribute to the secretion of pro-inflammatory cytokines, primarily IL-1β, IL-6, and TNFα, along with chemokines such as CCR (32). While this response is protective against pathogens, the swift production of pro-inflammatory cytokines typically stimulates the IIS, facilitating the effective elimination of pathogens. However, when self-tolerance is compromised, inflammation can evolve into a chronic and maladaptive immune response (33). Notably, CD80/CD86, co-stimulatory molecules found on these macrophages and other immune cells, respond to activation signals, which inhibit the proliferation of pathogens (34). In contrast to their pro-inflammatory counterparts, the phenotype of “anti-inflammatory” M2 macrophages is characterized by the expression of various surface markers, including macrophage scavenger receptors, mannose receptor-1, and the MER proto-oncogene tyrosine kinase (MerTK) (35). To fulfill their primary function in maintaining tissue homeostasis, these “alternative-activated” macrophages facilitate proliferation, contribute to bone and joint cartilage regeneration, and mitigate inflammatory processes (36). Their reparative functions include clearing apoptotic cells, synthesizing extracellular matrix (ECM) components, as well as promoting angiogenesis and chemokine activity. Additionally, IL-10 and TGF-β are produced endogenously by M2 macrophages, which helps steer immune activation toward a more favorable tissue repair process (37).

**Figure 3:**
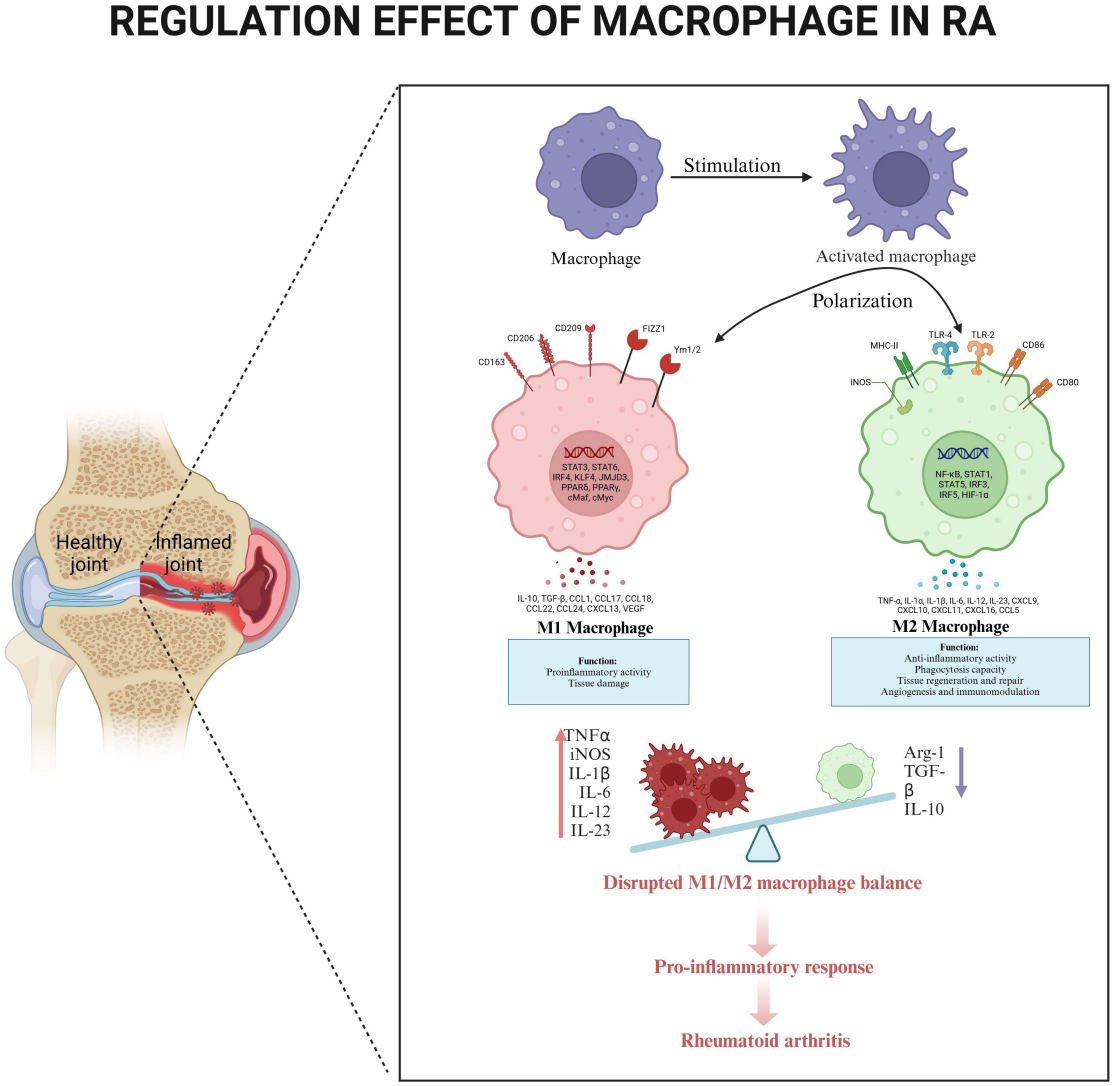
The regulatory effect of macrophages in RA. Circulating monocytes differentiate into synovial macrophages, which undergo activation and polarization in response to local inflammatory signals. Compares healthy joint homeostasis (left) with RA-inflamed joint pathology (right), highlighting characteristic synovial hyperplasia. M1 polarization dominates in RA, producing pro-inflammatory mediators (TNF-α, iNOS, IL-1β, IL-6, IL-12, IL-23) that promote tissue damage and osteoclast activation. An imbalance between M1 and M2 creates a self-sustaining inflammatory cascade, maintaining synovitis and joint destruction.

The clinical relevance of macrophage polarization is evident in RA progression. Macrophages are prevalent in synovial tissue during the active phase of RA, and their numbers decrease upon the attainment of clinical remission. This observation indicates that macrophages possess considerable plasticity, allowing them to respond to stimuli from their microenvironment, often through a process referred to as polarization. Importantly, numerous studies have indicated that alterations in the quantity of macrophages present in synovial tissue can serve as predictors of treatment outcomes. Notably, an imbalance in the subpopulations of macrophages has been observed in the synovial fluid of RA patients, who demonstrate a higher M1/M2 ratio in comparison to individuals with osteoarthritis (OA) (38), highlighting the pathological consequences of disrupted macrophage homeostasis. (**Figure 3**).

## 3 Regulation of neutrophils in RA

Neutrophils represent the most abundant circulating leukocytes and serve as primary responders in RA synovitis. As the most abundant circulating leukocytes, neutrophils serve as first responders in RA pathogenesis. Comprising ~60% of peripheral white blood cells, they rapidly infiltrate injured tissues and express immune-modulating molecules including cytokines, chemokines, and MHCI antigens (39). This functional versatility stems from rapid gene expression changes during inflammatory activation (40). While circulating neutrophils typically undergo apoptosis within 24 hours, synovial microenvironmental alterations in RA significantly prolong their lifespan and enhance effector functions (41). The sustained inflammatory milieu contains chemokines that regulate neutrophil chemotaxis and activation alongside other innate immune cells (42).

Chemokine networks orchestrate neutrophil recruitment in RA. Murayama et al. demonstrated that chemokine receptor interactions mediate leukocyte trafficking and synovitis, with broad-spectrum inhibition showing superior efficacy to single-target approaches due to pathway redundancy (43). This chemokine-driven recruitment upregulates neutrophil gene expression, modifying membrane receptor profiles and apoptosis regulation to create long-lived inflammatory neutrophils (44).

Pathogenic neutrophil activities in RA involve multiple synergistic mechanisms: enhanced migration, prolonged survival, oxidative stress responses, and neutrophil extracellular trap (NET) release (45). Notably, NETs have emerged as promising biomarkers, with plasma cell-free nucleosomes demonstrating high diagnostic specificity for early arthritis (46). Synovial infiltration engages adhesion molecules (P/E-selectins, integrin α2β), chemokine receptors (CXCR2, CCR1/2), and lipid mediators (LTB4) (47, 48). Upon activation, neutrophils dynamically adjust receptor expression to amplify synovial migration and release inflammatory mediators (cytokines, prostaglandins, leukotrienes) that perpetuate joint inflammation (49).

Anti-citrullinated protein antibodies (ACPAs) directly prime neutrophils toward pro-inflammatory states, increasing ROS production and upregulating MIP-1, IL-8, and IL-23 *in vitro* (50, 51). Synovial neutrophils further exacerbate inflammation through IL-17B expression, which synergizes with TNF-α to enhance leukocyte migration (52). The emerging role of IL-20 as a pro-inflammatory cytokine expressed by neutrophils and other synovial cells correlates with disease activity and ACPA positivity (53, 54).

Neutrophil-derived enzymes drive joint destruction through multiple pathways. Myeloperoxidase (MPO), abundant in azurophilic granules, contributes to RA pathology through NET formation and T-cell activation (55, 56). Similarly, neutrophil elastase (NE) degrades cartilage components (elastin, collagen) and activates PAR2-mediated p44/42 MAPK signaling to promote joint injury (57, 58). Oxidative stress is amplified by NADPH oxidase activity, which generates superoxide anions through p47phox phosphorylation, elevating ROS levels that exacerbate tissue damage (59, 60).

## 4 Regulation of complement in RA

The complement system’s dual role in protection and pathology is particularly relevant in RA. The complement system constitutes a fundamental component of the immune system, playing a pivotal role in various protective immune processes (61). These processes encompass the management and clearance of circulating immune complexes, recognition of foreign antigens, regulation of both humoral and cellular immunity, clearance of apoptotic and necrotic cells, and participation in injury resolution and tissue regeneration (62). However, in the context of RA, insufficient regulation of complement activation may contribute to the pathogenesis of numerous inflammatory and autoimmune diseases in humans, including RA, which adversely affects cartilage, bone, and synovium. Evidence supporting this link includes the identification of complement deposition within affected tissues, diminished levels of complement proteins in the bloodstream and/or synovial fluid of patients, elevated levels of complement activation fragments, and findings from experimental models (63). A prime example of this dysregulation comes from Sjöberg et al. who demonstrated that fibromodulin (FM), a cartilage extracellular matrix component, binds to C1q, activating the classical complement pathway while recruiting factor H (FH) to limit membrane attack complex (MAC) formation and C5a release, indicating a dual role in joint inflammation. The study revealed that FM’s complement activation occurs through ionic interactions with C1q, independent of its keratan sulfate chains. Conversely, FH binding to FM reduces terminal pathway activation, implicating FM as a potential factor in sustained arthritis inflammation (64).

The transition from physiological protection to pathological damage involves multiple complement functions. The essential functions of the complement system comprise the elimination of foreign microorganisms through specific recognition, regulation, and lysis. Additionally, the system is instrumental in the clearance of circulating immune complexes (CICs), apoptotic cells, apoptotic bodies, and necrotic cells (65). Of particular relevance to RA, among the various types of CICs—small, medium, and large—medium-sized CICs are typically implicated in the majority of tissue damage, as they tend to become entrapped within tissues or joints (66). These protective functions offer significant advantages to the host, even in the absence of adaptive immune responses. Under normal conditions, the complement system regulates its pro-inflammatory and anti-inflammatory functions through various inhibitors under normal physiological conditions (67). However, these natural complement inhibitors may become insufficient when the system is excessively activated during acute inflammatory episodes, potentially resulting in adverse effects. Furthermore, the functionality of the complement system extends beyond serum or plasma; it encompasses every tissue or organ within the body that may act as a direct target for different complement components (68).

Complement activation in RA occurs through three interconnected pathways. Most proteins of the complement system are typically found in circulation in an inactive (zymogen) state and are activated via proteolytic processes upon the recognition of danger signals (69). Notably, the complement system possesses multiple activation pathways, each employing distinct recognition molecules, which underscores the system’s complexity. Complement activation may occur through one of three pathways: the classical, lectin, or alternative pathways (70). These pathways converge to produce the activation of the complement system facilitates pathogen removal by opsonizing pathogens through surface deposition of complement component C3b, enhancing immune cell chemotaxis via the generation of anaphylatoxins such as complement C5a, and directly disrupting pathogen surfaces through the formation of the MAC C5b-C9 (71). In the rheumatoid joint, complement-mediated processes are fundamental to inflammation and may transpire independently of infection; thus, the inhibition of complement, such as through the blockade of C5a, has long been regarded as possessing therapeutic potential (72).

Therapeutic targeting of complement in RA requires careful consideration. Assessing the role of complement inhibition in RA presents considerable challenges. Complement is one of many inflammatory mediators involved in the pathogenesis of complex diseases like RA (73). While promising, numerous approved and highly effective treatment regimens for RA include complement inhibitors. Integrating complement inhibition within the expanding repertoire of effective treatment options necessitates careful deliberation. Moreover, complement deficiencies—particularly those affecting classical pathway components like C1q—predispose individuals to systemic lupus erythematosus (SLE), suggesting that patients with this condition should avoid medications that inhibit the activation and function of the classical pathway (74). In RA specifically, a potential trigger for complement activation may stem from immune complexes containing RA-associated antibodies. Approximately 60% of early RA patients demonstrate positivity for autoantibodies, such as ACPAs and/or rheumatoid factors (75). These antibodies interact with antigens within synovial joints, leading to the formation of immune complexes and subsequent local complement activation. Additionally, molecules released from the ECM of chondrocytes—including fibromodulin, osteomodulin, chondroadherin, the G3 domain of aggrecan, and cartilage oligomeric matrix protein (COMP)—serve as potent activators of the complement system (76).

## 5 Regulation of dendritic cells in RA

Dendritic cells (DCs) are fundamental members of the IIS whose importance in rheumatoid arthritis pathogenesis has become increasingly apparent. Since their discovery in 1973, our understanding of their crucial role in innate immune responses has expanded significantly (77). These specialized antigen-presenting cells provide a critical immunological bridge between innate and adaptive immunity through their unique capacity to continuously monitor the microenvironment while capturing and processing antigens (78). The clinical relevance of DCs in RA is underscored by recent findings demonstrating that the proportion of precursor DCs (pre-DCs) in peripheral blood shows strong correlation with treatment resistance, with their gene markers proving more predictive of therapeutic efficacy than established clinical parameters including ACPA status and disease duration (79). This growing body of evidence positions DCs as both valuable biomarkers and promising therapeutic targets in RA management.

The human DC compartment comprises several functionally distinct subsets that contribute differentially to RA pathogenesis. Conventional DCs (cDCs), identified by their CD11c expression, can be further subdivided into cDC1 and cDC2 populations. The cDC1 subset expresses high levels of CD141 and specializes in CD8+ T cell activation through MHC class I cross-presentation (80), while cDC2 cells, characterized by prominent CD1c expression, predominantly activate CD4+ T cells (81). Plasmacytoid DCs (pDCs), which express CD123, represent another major subset that responds to viral infections through rapid production of type I interferons (IFNs) and other cytokines (82). Beyond these classical populations, monocyte-derived DCs (moDCs) have emerged as important players in RA synovitis. These inflammatory DCs, which retain CD14 expression, are capable of inducing naïve CD4+ T cell differentiation into Th17 cells and promoting synovitis development (83, 84). Conversely, exposure to specific growth factors, cytokines or pharmaceutical agents can promote monocyte differentiation into tolerogenic DCs (tOLDCs), suggesting potential avenues for therapeutic intervention (85).

The functional state of DCs, particularly their maturation status, profoundly influences their immunological impact in RA. Mature DCs differ markedly from their immature counterparts in their ability to secrete copious amounts of cytokines and activate diverse antigen-specific T lymphocyte populations (including Th1, Th2, Th17 and Treg cells) within secondary lymphoid organs (86, 87). This maturation process is driven by recognition of PAMPs, DAMPs, and various inflammatory cytokines (88). During maturation, DCs undergo significant phenotypic changes, upregulating surface expression of CD80, CD86, and MHC-II while downregulating phagocytic capacity - modifications that facilitate productive interactions with T cells (89). In the rheumatoid joint, this maturation process contributes to disease progression through multiple mechanisms. Activated DCs not only initiate and stimulate T cell responses that drive local and systemic inflammation, but also secrete a wide array of inflammatory mediators that promote innate immune cell activation. Furthermore, they facilitate the development of ectopic lymphoid structures within affected joints (90). Of particular relevance to RA, collagen II has been shown to potently induce DC maturation, while the resulting mature DCs reciprocally enhance collagen degradation in joint tissues - establishing a self-perpetuating cycle that accelerates joint destruction (91). The diverse DC subsets each exhibit distinct functional and phenotypic characteristics in RA, with their altered behavior intimately linked to disease pathogenesis.

## 6 Regulation of mast cell in RA

Emerging evidence highlights the pivotal yet complex role of mast cells (MCs) in rheumatoid arthritis pathogenesis, where they function as dynamic immunomodulators at the interface of innate and adaptive immunity. MCs, originating from the hematopoietic system, are innate immune cells that serve as one of the most crucial sentinels for detecting danger signals; their main role bridges the gap between the IIS and AIS (92). While their involvement in IgE-mediated allergic inflammation is well-documented, recent studies reveal their equally important participation in non-allergic inflammatory processes, acting as an early warning system against invaders and coordinating immune responses (93). Within the rheumatoid joint, MCs are widely distributed in tissues directly exposed to the external environment, and upon activation, they rapidly degranulate, releasing a plethora of preformed mediators and initiating the resynthesis of other mediators, including proteases, growth factors, cytokines, and chemokines (94). Although increased MC infiltration in the synovium has been consistently reported in RA, the exact nature of their involvement - whether as active participants or passive bystanders - remains a subject of ongoing investigation.

The extensive mediator repertoire of MCs underscores their multifaceted role in RA pathophysiology. MCs secrete diverse products, including proteases (tryptase, chymase), proteoglycans (heparin), biogenic amines (histamine), growth factors (VEGF, PDGF), and an array of cytokines (TNFα, IL-1β, IL-6, IL-17) (95). Notably, their ability to synthesize and release lipid mediators like PGD2 and LTB4 adds another layer of complexity to their inflammatory modulation, with degranulation serving as the key regulatory point for mediator release (96). In the context of RA, this degranulation process differs from allergic responses, relying instead on specific interactions between VAMP8 and VAMP7 on MC granule membranes (97).

The immunoregulatory network involving MCs extends to their dynamic crosstalk with other immune cells. The interplay between regulatory T cells (T-regs) and MCs represents a particularly intriguing pseudosymbiotic relationship, where T-regs attract mast cells through IL-9 secretion, while MCs reciprocally support T-reg proliferation via IL-2 release (98). Beyond this, MCs demonstrate remarkable functional plasticity, serving as antigen-presenting cells capable of activating T cells while also influencing B cell differentiation and antibody production (99). This multifaceted interactivity enables MCs to exert both pro-inflammatory and anti-inflammatory effects, though the precise mechanisms governing this duality remain incompletely understood (100).

In the synovial microenvironment, MCs participate actively in joint destruction through multiple pathways. Human synovial MCs are frequently found in proximity to monocytes/macrophages, T cells, and B cells (101), where they promote inflammation by enhancing vascular permeability and secreting chemokines like IL-8 that drive neutrophil infiltration (102). Furthermore, MC-derived mediators such as TNFα, IL-1, and IL-17 activate synovial fibroblasts, while trypsin released by MCs activates MMPs that degrade cartilage extracellular matrix components (103, 104). The impact of MCs extends to bone remodeling, where they may accelerate bone turnover through both direct effects on osteoclasts and indirect cytokine-mediated mechanisms (105, 106).

Despite strong evidence supporting a pro-inflammatory role in RA, MCs can also exhibit protective functions depending on microenvironmental cues. While they can exacerbate inflammation through TNF-α production (synergistically enhanced by IL-33), they can also suppress immune responses via IL-10 and histamine release when activated by immune complexes (107, 108). This functional dichotomy highlights the need for careful therapeutic targeting of MCs in RA, as their complex biology demands strategies that can selectively modulate their detrimental effects while preserving beneficial functions. Future research should focus on elucidating the contextual factors that determine MC phenotype and function in RA, paving the way for more precise therapeutic interventions.

## 7 Regulation of natural killer cells in RA

Natural killer (NK) cells represent a critical component of the innate immune system that plays complex and multifaceted roles in RA pathogenesis. Initially discovered in the 1970s as large granular lymphocytes derived from common lymphoid progenitors, NK cells were first recognized for their potent cytotoxicity against virus-infected cells and tumors (109). Subsequent research has significantly expanded our understanding of NK cell biology, revealing their classification as innate lymphoid cells capable of bridging innate and adaptive immunity through diverse effector functions (110). These unique lymphocytes lack T-cell or B-cell receptors and are identified by CD3 negativity, but can be divided into functionally distinct subsets based on CD16 and CD56 expression patterns. The CD16+CD56dim subset specializes in cytotoxic functions, while CD16-CD56bright cells produce cytokines similar to CD4+ T helper cells, and a regulatory NK subset producing IL-10 contributes to immune regulation (111). The activation of NK cells represents a precisely balanced process governed by the interplay of surface activating and inhibitory receptors, with additional modulation by cytokines including IL-2, IL-12, IL-15, and IL-18 (112). Through both direct cytotoxicity and cytokine production, NK cells participate in the immune dysregulation characteristic of RA, though their precise role at the interface of innate and adaptive immunity requires further investigation (113).

The functional diversity of NK cells extends to their developmental biology and tissue-specific activities. Since their discovery, extensive research has characterized NK cell subsets with distinct developmental origins, anatomical distributions, and effector capabilities (114). These include not only natural cytotoxicity and antibody-dependent cellular cytotoxicity (ADCC), but also the production of diverse cytokines that mirror the functional specialization of CD4+ T helper subsets (114). Emerging evidence highlights an important regulatory role for NK cells through IL-10 production and selective targeting of autoreactive cells, suggesting their involvement in maintaining immune homeostasis (114). In the context of RA, both cytotoxic mechanisms and cytokine production by NK cells contribute to disease modulation, though their net effect remains controversial.

NK cells exhibit compartmentalized changes in RA that reflect their complex involvement in disease pathogenesis. While accumulating in the synovial fluid of RA patients (115), the causal relationship between NK cell infiltration and disease development remains unclear. Animal models yield seemingly contradictory results, with NK cell depletion exacerbating collagen-induced arthritis in some studies (116) while ameliorating disease in others (117). Human studies demonstrate a redistribution of NK cells from peripheral blood to synovial tissue, with decreased circulating NK cells but increased synovial infiltration, particularly of activated subsets (118, 119). This spatial redistribution suggests active recruitment to inflamed joints, where NK cells likely interact with other immune cells to influence disease progression (120). Notably, synovial NK cells demonstrate altered functional capacity, promoting osteoclast differentiation when co-cultured with monocytes (121) while potentially also lysing autoreactive immune cells (122). The CD56 bright subset appears particularly relevant in RA synovium (123), where it may drive inflammation through production of pro-inflammatory cytokines like TNF-α and IFN-γ (124). These cytokines not only perpetuate local inflammation but also promote dendritic cell maturation and lymphocyte activation, creating self-amplifying inflammatory loops (125). Paradoxically, NK cells may also exert protective effects by suppressing Th17 differentiation and osteoclastogenesis through IFN-γ production (126), illustrating their functional duality in RA.

Multiple lines of evidence point to NK cell dysfunction in RA patients. Compared to healthy controls, RA patients demonstrate reduced NK cell cytotoxic activity (127) and decreased frequencies of perforin-positive NK cells (128). Despite these advances, the precise role of NK cells in RA pathogenesis remains incompletely understood. Current evidence suggests their impact may be context-dependent, varying according to disease stage, microenvironmental cues, and the specific NK subset involved (129, 130). A more comprehensive understanding of NK cell biology in the context of inflammatory arthritis will be essential for developing targeted therapeutic strategies to modulate their activity in RA.

## 8 Therapeutic strategies and future perspectives

RA remains a challenging chronic inflammatory disorder characterized by persistent synovitis that, when inadequately controlled, progresses to irreversible joint damage and functional impairment. While the implementation of treat-to-target strategies and biologic therapies has significantly improved patient outcomes, substantial therapeutic gaps persist, particularly for those failing to achieve remission or developing refractory disease despite available treatments. This unmet clinical need underscores the necessity for novel therapeutic approaches targeting alternative pathogenic pathways.

The current therapeutic arsenal for RA relies heavily on disease-modifying antirheumatic drugs (DMARDs), with methotrexate (MTX) maintaining its position as the cornerstone therapy due to its well-established antimetabolite activity. However, the limitations of MTX are apparent, as approximately half of patients show insufficient radiographic improvement, driving the search for more effective alternatives. This therapeutic shortfall has been partially addressed by the development of biologic DMARDs, with ten currently approved by the U.S. FDA, most targeting key inflammatory mediators like TNF (Remicade) and IL-6 (Actemra). Nevertheless, about 30% of patients exhibit either primary non-response or secondary loss of efficacy to these biologics, highlighting the complexity of RA pathogenesis and the involvement of additional mechanisms beyond current therapeutic targets.

Emerging therapeutic strategies are increasingly focusing on innate immune pathways, as systematically outlined in **Table 1**. Notable developments include NK cell-targeted approaches such as the CD16/CD30 bispecific antibody AFM13 (Phase II) and IL-15/IL-2 cytokine therapy (Phase I) (131, 132), macrophage-directed interventions featuring CSF1R inhibition (Phase II) and JAK inhibitor-mediated polarization (clinically approved) (133), and complement system blockade via anti-C5a (Phase III) and C1q inhibitors (preclinical) (64, 135).

**Table 1:** Current medications and clinical trials targeting innate immune cells in RA.

Target/cell type	Drug/therapy	Mechanism	Development stage	References
NK Cells	AFM13 (CD16/CD30 bispecific)	Phase II (lymphoma; RA potential)	Phase II (lymphoma; RA potential)	(131)
	IL-15/IL-2 cytokine therapy	Boosts NK cell proliferation/activation	Preclinical/Phase I (RA)	(132)
Macrophages	Anti-CSF1R (e.g., emactuzumab)	Depletes pro-inflammatory macrophages	Phase II (RA)	(133)
	JAK inhibitors (tofacitinib)	Modulates macrophage polarization	Approved (RA)	(134)
Complement System	Anti-C5a (avacopan)	Blocks C5aR to reduce inflammation	Phase III (ANCA vasculitis; RA trials pending)	(64)
	C1q inhibitors (ANX-M1)	Inhibits the classical complement pathway	Preclinical (RA)	(135)
Combination Therapies	CAR-NK + anti-TNFα	Genetically modified NK cells + cytokine blockade	Phase I (RA)	(136)

Particularly promising are first-generation combination therapies like CAR-NK with anti-TNFα (Phase I), which demonstrate synergistic modulation of both innate and adaptive immune responses. While these advances reflect growing recognition of innate immunity’s role in RA, clinical translation still lags behind adaptive immune-targeted biologics, with challenges including optimization of cell-based delivery systems and overcoming the immunosuppressive synovial microenvironment. (**Table 1**).

The future therapeutic landscape of RA is rapidly expanding beyond conventional approaches, with several innovative strategies under investigation. These include selective JAK inhibition (with next-generation inhibitors in development), modulation of the GM-CSF pathway, targeting of Bruton’s tyrosine kinase (BTK, currently in phase II trials), manipulation of the PI3K pathway, neural modulation approaches, and dendritic cell-based therapies. This diversification of therapeutic options promises to yield more balanced treatment paradigms that integrate biological DMARDs with targeted small-molecule therapies, potentially addressing the current limitations in RA management.

## References

[1] Brown P Pratt AG Hyrich KL. Therapeutic advances in rheumatoid arthritis. BMJ. (2024) 384:e070856. doi: 10.1136/bmj-2022-070856, PMID: 38233032

[2] Radu AF Bungau SG. Management of rheumatoid arthritis: an overview. Cells. (2021) 10:2857. doi: 10.3390/cells10112857, PMID: 34831081 PMC8616326

[3] Jang S Kwon EJ Lee JJ. Rheumatoid arthritis: pathogenic roles of diverse immune cells. Int J Mol Sci. (2022) 23:905. doi: 10.3390/ijms23020905, PMID: 35055087 PMC8780115

[4] Ngo ST Steyn FJ McCombe PA. Gender differences in autoimmune disease. Front Neuroendocrinol. (2014) 35:347–69. doi: 10.1016/j.yfrne.2014.04.004, PMID: 24793874

[5] Kim H Cho SK Kim JW Jung SY Jang EJ Bae SC. An increased disease burden of autoimmune inflammatory rheumatic diseases in Korea. Semin Arthritis Rheumatol. (2020) 50:526–33. doi: 10.1016/j.semarthrit.2019.11.007, PMID: 31852583

[6] Di Matteo A Bathon JM Emery P. Rheumatoid arthritis. Lancet. (2023) 402:2019–33. doi: 10.1016/S0140-6736(23)01525-8, PMID: 38240831

[7] Hillion S Arleevskaya MI Blanco P Bordron A Brooks WH Cesbron JY. The innate part of the adaptive immune system. Clin Rev Allergy Immunol. (2020) 58:151–4. doi: 10.1007/s12016-019-08740-1, PMID: 31154567

[8] Hato T Dagher PC. How the innate immune system senses trouble and causes trouble. Clin J Am Soc Nephrol. (2015) 10:1459–69. doi: 10.2215/CJN.04680514, PMID: 25414319 PMC4527020

[9] Makuch S Więcek K Woźniak M. The immunomodulatory and anti-inflammatory effect of curcumin on immune cell populations, cytokines, and *in vivo* models of rheumatoid arthritis. Pharm (Basel). (2021) 14:309. doi: 10.3390/ph14040309, PMID: 33915757 PMC8065689

[10] Cooper MD Herrin BR. How did our complex immune system evolve? Nat Rev Immunol. (2010) 10:2–3. doi: 10.1038/nri2686, PMID: 20039476

[11] Elshabrawy HA Chen Z Volin MV Ravella S Virupannavar S Shahrara S. The pathogenic role of angiogenesis in rheumatoid arthritis. Angiogenesis. (2015) 18:433–48. doi: 10.1007/s10456-015-9477-2, PMID: 26198292 PMC4879881

[12] Mukhopadhyay M. The human immune interactome. Nat Methods. (2022) 19:1166. doi: 10.1038/s41592-022-01649-2, PMID: 36198837

[13] Wu X Yang ZH Wu J Han J. Ribosome-rescuer PELO catalyzes the oligomeric assembly of NOD-like receptor family proteins via activating their ATPase enzymatic activity. Immunity. (2023) 56:926–943.e7. doi: 10.1016/j.immuni.2023.02.014, PMID: 36948192

[14] Alivernini S MacDonald L Elmesmari A Finlay S Tolusso B Gigante MR. Distinct synovial tissue macrophage subsets regulate inflammation and remission in rheumatoid arthritis. Nat Med. (2020) 26:1295–306. doi: 10.1038/s41591-020-0939-8, PMID: 32601335

[15] Zaiss MM Joyce Wu HJ Mauro D Schett G Ciccia F. The gut-joint axis in rheumatoid arthritis. Nat Rev Rheumatol. (2021) 17:224–37. doi: 10.1038/s41584-021-00585-3, PMID: 33674813

[16] Hensvold A Klareskog L. Towards prevention of autoimmune diseases: The example of rheumatoid arthritis. Eur J Immunol. (2021) 51:1921–33. doi: 10.1002/eji.202048952, PMID: 34110013

[17] Walmsley M Butler DM Marinova-Mutafchieva L Feldmann M. An anti-inflammatory role for interleukin-11 in established murine collagen-induced arthritis. Immunology. (1998) 95:31–7. doi: 10.1046/j.1365-2567.1998.00568, PMID: 9767454 PMC1364373

[18] Tak PP Hart BA Kraan MC Jonker M Smeets TJ Breedveld FC. The effects of interferon beta treatment on arthritis. Rheumatol (Oxford). (1999) 38:362–9. doi: 10.1093/rheumatology/38.4.362, PMID: 10378715

[19] van Holten J Reedquist K Sattonet-Roche P Smeets TJ Plater-Zyberk C Vervoordeldonk MJ. Treatment with recombinant interferon-beta reduces inflammation and slows cartilage destruction in the collagen-induced arthritis model of rheumatoid arthritis. Arthritis Res Ther. (2004) 6:R239–49. doi: 10.1186/ar1165, PMID: 15142270 PMC416442

[20] Chen Z Bozec A Ramming A Schett G. Anti-inflammatory and immune-regulatory cytokines in rheumatoid arthritis. Nat Rev Rheumatol. (2019) 15:9–17. doi: 10.1038/s41584-018-0109-2, PMID: 30341437

[21] Jager NA Teteloshvili N Zeebregts CJ Westra J Bijl M. Macrophage folate receptor-β (FR-β) expression in auto-immune inflammatory rheumatic diseases: a forthcoming marker for cardiovascular risk? Autoimmun Rev. (2012) 11:621–6. doi: 10.1016/j.autrev.2011.11.002, PMID: 22094710

[22] Guo Q Wang Y Xu D Nossent J Pavlos NJ Xu J. Rheumatoid arthritis: pathological mechanisms and modern pharmacologic therapies. Bone Res. (2018) 6:15. doi: 10.1038/s41413-018-0016-9, PMID: 29736302 PMC5920070

[23] Smolen JS Aletaha D Barton A Burmester GR Emery P Firestein GS. Rheumatoid arthritis. Nat Rev Dis Primers. (2018) 4:18001. doi: 10.1038/nrdp.2018.1, PMID: 29417936

[24] Bartok B Firestein GS. Fibroblast-like synoviocytes: key effector cells in rheumatoid arthritis. Immunol Rev. (2010) 233:233–55. doi: 10.1111/j.0105-2896.2009.00859, PMID: 20193003 PMC2913689

[25] Shapouri-Moghaddam A Mohammadian S Vazini H Taghadosi M Esmaeili SA Mardani F. Macrophage plasticity, polarization, and function in health and disease. J Cell Physiol. (2018) 233:6425–40. doi: 10.1002/jcp.26429, PMID: 29319160 PMC13482560

[26] Quero L Hanser E Manigold T Tiaden AN Kyburz D. TLR2 stimulation impairs anti-inflammatory activity of M2-like macrophages, generating a chimeric M1/M2 phenotype. Arthritis Res Ther. (2017) 19:245. doi: 10.1186/s13075-017-1447-1, PMID: 29096690 PMC5667453

[27] Ginhoux F Schultze JL Murray PJ Ochando J Biswas SK. New insights into the multidimensional concept of macrophage ontogeny, activation and function. Nat Immunol. (2016) 17:34–40. doi: 10.1038/ni.3324, PMID: 26681460

[28] Martinez FO Gordon S. The M1 and M2 paradigm of macrophage activation: time for reassessment. F1000Prime Rep. (2014) 6:13. doi: 10.12703/P6-13, PMID: 24669294 PMC3944738

[29] Cutolo M Soldano S Gotelli E Montagna P Campitiello R Paolino S. CTLA4-Ig treatment induces M1-M2 shift in cultured monocyte-derived macrophages from healthy subjects and rheumatoid arthritis patients. Arthritis Res Ther. (2021) 23:306. doi: 10.1186/s13075-021-02691-9, PMID: 34952630 PMC8709961

[30] Gao Y Xu X Zhang X. Targeting different phenotypes of macrophages: A potential strategy for natural products to treat inflammatory bone and joint diseases. Phytomedicine. (2023) 118:154952. doi: 10.1016/j.phymed.2023.154952, PMID: 37506402

[31] Yunna C Mengru H Lei W Weidong C. Macrophage M1/M2 polarization. Eur J Pharmacol. (2020) 877:173090. doi: 10.1016/j.ejphar.2020.173090, PMID: 32234529

[32] Locati M Curtale G Mantovani A. Diversity, mechanisms, and significance of macrophage plasticity. Annu Rev Pathol. (2020) 15:123–47. doi: 10.1146/annurev-pathmechdis-012418-012718, PMID: 31530089 PMC7176483

[33] Netea MG Domínguez-Andrés J Barreiro LB Chavakis T Divangahi M Fuchs E. Defining trained immunity and its role in health and disease. Nat Rev Immunol. (2020) 20:375–88. doi: 10.1038/s41577-020-0285-6, PMID: 32132681 PMC7186935

[34] Ma C Wang C Zhang Y Li Y Fu K Gong L. Phillygenin inhibited M1 macrophage polarization and reduced hepatic stellate cell activation by inhibiting macrophage exosomal miR-125b-5p. BioMed Pharmacother. (2023) 159:114264. doi: 10.1016/j.biopha.2023.114264, PMID: 36652738

[35] Zhang J Muri J Fitzgerald G Gorski T Gianni-Barrera R Masschelein E. Endothelial lactate controls muscle regeneration from ischemia by inducing M2-like macrophage polarization. Cell Metab. (2020) 31:1136–1153.e7. doi: 10.1016/j.cmet.2020.05.004, PMID: 32492393 PMC7267778

[36] Huang X Wang X Ma L Wang H Peng Y Liu H. M2 macrophages with inflammation tropism facilitate cementoblast mineralization. J Periodontol. (2023) 94:290–300. doi: 10.1002/JPER.22-0048, PMID: 35912930

[37] Tardito S Martinelli G Soldano S Paolino S Pacini G Patane M. Macrophage M1/M2 polarization and rheumatoid arthritis: A systematic review. Autoimmun Rev. (2019) 18:102397. doi: 10.1016/j.autrev.2019.102397, PMID: 31520798

[38] Zhang H Cai D Bai X. Macrophages regulate the progression of osteoarthritis. Osteoarthritis Cartilage. (2020) 28:555–61. doi: 10.1016/j.joca.2020.01.007, PMID: 31982565

[39] Yuan K Zhu Q Lu Q Jiang H Zhu M Li X. Quercetin alleviates rheumatoid arthritis by inhibiting neutrophil inflammatory activities. J Nutr Biochem. (2020) 84:108454. doi: 10.1016/j.jnutbio.2020.108454, PMID: 32679549

[40] Beck DB Ferrada MA Sikora KA Ombrello AK Collins JC Pei W. Somatic mutations in UBA1 and severe adult-onset autoinflammatory disease. N Engl J Med. (2020) 383:2628–38. doi: 10.1056/NEJMoa2026834, PMID: 33108101 PMC7847551

[41] Liew PX Kubes P. The neutrophil’s role during health and disease. Physiol Rev. (2019) 99:1223–48. doi: 10.1152/physrev.00012, PMID: 30758246

[42] Zhao W Zhao H Li M Huang C. Microfluidic devices for neutrophil chemotaxis studies. J Transl Med. (2020) 18:168. doi: 10.1186/s12967-020-02335-7, PMID: 32293474 PMC7158383

[43] Murayama MA Shimizu J Miyabe C Yudo K Miyabe Y. Chemokines and chemokine receptors as promising targets in rheumatoid arthritis. Front Immunol. (2023) 14:1100869. doi: 10.3389/fimmu.2023.1100869, PMID: 36860872 PMC9968812

[44] Aga E Mukherjee A Rane D More V Patil T van Zandbergen G. Type-1 interferons prolong the lifespan of neutrophils by interfering with members of the apoptotic cascade. Cytokine. (2018) 112:21–6. doi: 10.1016/j.cyto.2018.06.027, PMID: 30554594

[45] Kurowska W Kuca-Warnawin EH Radzikowska A Maśliński W. The role of anti-citrullinated protein antibodies (ACPA) in the pathogenesis of rheumatoid arthritis. Cent Eur J Immunol. (2017) 42:390–8. doi: 10.5114/ceji.2017.72807, PMID: 29472818 PMC5820977

[46] Rogers E Pothugunta S Kosmider V Stokes N Bonomini L Briggs GD. The diagnostic, therapeutic and prognostic relevance of neutrophil extracellular traps in polytrauma. Biomolecules. (2023) 13:1625. doi: 10.3390/biom13111625, PMID: 38002307 PMC10669581

[47] O’Neil LJ Oliveira CB Wang X Navarrete M Barrera-Vargas A Merayo-Chalico J. Neutrophil extracellular trap-associated carbamylation and histones trigger osteoclast formation in rheumatoid arthritis. Ann Rheum Dis. (2023) 82:630–8. doi: 10.1136/ard-2022-223568, PMID: 36737106 PMC11302494

[48] Kim IS Kim YS Jang SW Sung HJ Han KH Na DS. Differential effects of 9-cis retinoic acid on expression of CC chemokine receptors in human monocytes. Biochem Pharmacol. (2004) 68:611–20. doi: 10.1016/j.bcp.2004.03.041, PMID: 15276068

[49] Thieblemont N Wright HL Edwards SW Witko-Sarsat V. Human neutrophils in auto-immunity. Semin Immunol. (2016) 28:159–73. doi: 10.1016/j.smim.2016.03.004, PMID: 27036091

[50] Wu CY Yang HY Lai JH. Anti-citrullinated protein antibodies in patients with rheumatoid arthritis: biological effects and mechanisms of immunopathogenesis. Int J Mol Sci. (2020) 21:4015. doi: 10.3390/ijms21114015, PMID: 32512739 PMC7312469

[51] Coutant F. Pathogenic effects of anti-citrullinated protein antibodies in rheumatoid arthritis - role for glycosylation. Joint Bone Spine. (2019) 86:562–7. doi: 10.1016/j.jbspin.2019.01.005, PMID: 30685537

[52] Khandpur R Carmona-Rivera C Vivekanandan-Giri A Gizinski A Yalavarthi S Knight JS. NETs are a source of citrullinated autoantigens and stimulate inflammatory responses in rheumatoid arthritis. Sci Transl Med. (2013) 5:178ra40. doi: 10.1126/scitranslmed.3005580, PMID: 23536012 PMC3727661

[53] Šenolt L Prajzlerová K Hulejová H Šumová B Filková M Veigl D. Interleukin-20 is triggered by TLR ligands and associates with disease activity in patients with rheumatoid arthritis. Cytokine. (2017) 97:187–92. doi: 10.1016/j.cyto.2017.06.009, PMID: 28662439

[54] Chokesuwattanaskul S Phelan MM Edwards SW Wright HL. A robust intracellular metabolite extraction protocol for human neutrophil metabolic profiling. PloS One. (2018) 13:e0209270. doi: 10.1371/journal.pone.0209270, PMID: 30571714 PMC6301625

[55] Strzepa A Pritchard KA Dittel BN. Myeloperoxidase: A new player in autoimmunity. Cell Immunol. (2017) 317:1–8. doi: 10.1016/j.cellimm.2017.05.002, PMID: 28511921 PMC5665680

[56] Shamsuzzaman S Deaton RA Salamon A Doviak H Serbulea V Milosek VM. Novel mouse model of myocardial infarction, plaque rupture, and stroke shows improved survival with myeloperoxidase inhibition. Circulation. (2024) 150:687–705. doi: 10.1161/CIRCULATIONAHA.123.067931, PMID: 38881440 PMC11347105

[57] Di Cesare Mannelli L Micheli L Cinci L Maresca M Vergelli C Pacini A. Effects of the neutrophil elastase inhibitor EL-17 in rat adjuvant-induced arthritis. Rheumatol (Oxford). (2016) 55:1285–94. doi: 10.1093/rheumatology/kew055, PMID: 27032424 PMC5009473

[58] Muley MM Krustev E McDougall JJ. Preclinical assessment of inflammatory pain. CNS Neurosci Ther. (2016) 22:88–101. doi: 10.1111/cns.12486, PMID: 26663896 PMC6492823

[59] Lee HR Yoo SJ Kim J Yoo IS Park CK Kang SW. The effect of nicotinamide adenine dinucleotide phosphate oxidase 4 on migration and invasion of fibroblast-like synoviocytes in rheumatoid arthritis. Arthritis Res Ther. (2020) 22:116. doi: 10.1186/s13075-020-02204-0, PMID: 32414400 PMC7227051

[60] Wu Q Zhong ZM Zhu SY Liao CR Pan Y Zeng JH. Advanced oxidation protein products induce chondrocyte apoptosis via receptor for advanced glycation end products-mediated, redox-dependent intrinsic apoptosis pathway. Apoptosis. (2016) 21:36–50. doi: 10.1007/s10495-015-1191-4, PMID: 26520447

[61] Zheng F Put S Bouwens L Lahoutte T Matthys P Muyldermans S. Molecular imaging with macrophage CRIg-targeting nanobodies for early and preclinical diagnosis in a mouse model of rheumatoid arthritis. J Nucl Med. (2014) 55:824–9. doi: 10.2967/jnumed.113.130617, PMID: 24686780

[62] Ricklin D Reis ES Lambris JD. Complement in disease: a defence system turning offensive. Nat Rev Nephrol. (2016) 12:383–401. doi: 10.1038/nrneph.2016.70, PMID: 27211870 PMC4974115

[63] Morgan BP Harris CL. Complement, a target for therapy in inflammatory and degenerative diseases. Nat Rev Drug Discov. (2015) 14:857–77. doi: 10.1038/nrd4657, PMID: 26493766 PMC7098197

[64] Sjöberg A Onnerfjord P Mörgelin M Heinegård D Blom AM. The extracellular matrix and inflammation: fibromodulin activates the classical pathway of complement by directly binding C1q. J Biol Chem. (2005) 280:32301–8. doi: 10.1074/jbc.M504828200, PMID: 16046396

[65] Zhao X Okeke NL Sharpe O Batliwalla FM Lee AT Ho PP. Circulating immune complexes contain citrullinated fibrinogen in rheumatoid arthritis. Arthritis Res Ther. (2008) 10:R94. doi: 10.1186/ar2478, PMID: 18710572 PMC2575608

[66] Zhao J Xu L Wei K Jiang P Chang C Xu L. Identification of clinical characteristics biomarkers for rheumatoid arthritis through targeted DNA methylation sequencing. Int Immunopharmacol. (2024) 131:111860. doi: 10.1016/j.intimp.2024.111860, PMID: 38508093

[67] Trouw LA Rispens T Toes REM. Beyond citrullination: other post-translational protein modifications in rheumatoid arthritis. Nat Rev Rheumatol. (2017) 13:331–9. doi: 10.1038/nrrheum.2017.15, PMID: 28275265

[68] Kwon EJ Ju JH. Impact of posttranslational modification in pathogenesis of rheumatoid arthritis: focusing on citrullination, carbamylation, and acetylation. Int J Mol Sci. (2021) 22:10576. doi: 10.3390/ijms221910576, PMID: 34638916 PMC8508717

[69] Trejo-Zambrano MI Gómez-Bañuelos E Andrade F. Redox-mediated carbamylation as a hapten model applied to the origin of antibodies to modified proteins in rheumatoid arthritis. Antioxid Redox Signal. (2022) 36:389–409. doi: 10.1089/ars.2021.0064, PMID: 33906423 PMC8982126

[70] Wang P Wu Q Shuai ZW. Emerging role of ficolins in autoimmune diseases. Pharmacol Res. (2021) 163:105266. doi: 10.1016/j.phrs.2020.105266, PMID: 33127557

[71] Banda NK Mehta G Ferreira VP Cortes C Pickering MC Pangburn MK. Essential role of surface-bound complement factor H in controlling immune complex-induced arthritis. J Immunol. (2013) 190:3560–9. doi: 10.4049/jimmunol.1203271, PMID: 23436934 PMC3607402

[72] Zouboulis CC Frew JW Giamarellos-Bourboulis EJ Jemec GBE Del Marmol V Marzano AV. Target molecules for future hidradenitis suppurativa treatment. Exp Dermatol. (2021) 30 Suppl 1:8–17. doi: 10.1111/exd.14338, PMID: 34085329

[73] Malmström V Catrina AI Klareskog L. The immunopathogenesis of seropositive rheumatoid arthritis: from triggering to targeting. Nat Rev Immunol. (2017) 17:60–75. doi: 10.1038/nri.2016.124, PMID: 27916980

[74] Frangou E Garantziotis P Grigoriou M Banos A Nikolopoulos D Pieta A. Cross-species transcriptome analysis for early detection and specific therapeutic targeting of human lupus nephritis. Ann Rheum Dis. (2022) 81:1409–19. doi: 10.1136/annrheumdis-2021-222069, PMID: 35906002 PMC9484391

[75] Zufferey P Rebell C Benaim C Ziswiler HR Dumusc A So A. Ultrasound can be useful to predict an evolution towards rheumatoid arthritis in patients with inflammatory polyarthralgia without anticitrullinated antibodies. Joint Bone Spine. (2017) 84:299–303. doi: 10.1016/j.jbspin.2016.05.011, PMID: 27369647

[76] Ziegelasch M Eloff E Hammer HB Cedergren J Martinsson K Reckner Å. Bone erosions detected by ultrasound are prognostic for clinical arthritis development in patients with ACPA and musculoskeletal pain. Front Med (Lausanne). (2021) 8:653994. doi: 10.3389/fmed.2021.653994, PMID: 33834034 PMC8021704

[77] Steinman RM Cohn ZA. Identification of a novel cell type in peripheral lymphoid organs of mice. I. Morphology, quantitation, tissue distribution. J Exp Med. (1973) 137:1142–62. doi: 10.1084/jem.137.5.1142, PMID: 4573839 PMC2139237

[78] Banchereau J Steinman RM. Dendritic cells and the control of immunity. Nature. (1998) 392:245–52. doi: 10.1038/32588, PMID: 9521319

[79] Wu L Dakic A. Development of dendritic cell system. Cell Mol Immunol. (2004) 1:112–8. 16212897

[80] Rana AK Li Y Dang Q Yang F. Monocytes in rheumatoid arthritis: Circulating precursors of macrophages and osteoclasts and, their heterogeneity and plasticity role in RA pathogenesis. Int Immunopharmacol. (2018) 65:348–59. doi: 10.1016/j.intimp.2018.10.016, PMID: 30366278

[81] Cavanagh LL Boyce A Smith L Padmanabha J Filgueira L Pietschmann P. Rheumatoid arthritis synovium contains plasmacytoid dendritic cells. Arthritis Res Ther. (2005) 7:R230–40. doi: 10.1186/ar1467, PMID: 15743469 PMC1065313

[82] Sun BY Wang ZT Chen KZ Song Y Wu JF Zhang D. Mobilization and activation of tumor-infiltrating dendritic cells inhibits lymph node metastasis in intrahepatic cholangiocarcinoma. Cell Death Discov. (2024) 10:304. doi: 10.1038/s41420-024-02079-z, PMID: 38926350 PMC11208581

[83] Harding B Knight SC. The distribution of dendritic cells in the synovial fluids of patients with arthritis. Clin Exp Immunol. (1986) 63:594–600. 2940038 PMC1577554

[84] Miossec P. Dynamic interactions between T cells and dendritic cells and their derived cytokines/chemokines in the rheumatoid synovium. Arthritis Res Ther. (2008) 10 Suppl 1:S2. doi: 10.1186/ar2413, PMID: 19007422 PMC2582809

[85] Merad M Sathe P Helft J Miller J Mortha A. The dendritic cell lineage: ontogeny and function of dendritic cells and their subsets in the steady state and the inflamed setting. Annu Rev Immunol. (2013) 31:563–604. doi: 10.1146/annurev-immunol-020711-074950, PMID: 23516985 PMC3853342

[86] Moret FM Hack CE van der Wurff-Jacobs KM de Jager W Radstake TR Lafeber FP. Intra-articular CD1c-expressing myeloid dendritic cells from rheumatoid arthritis patients express a unique set of T cell-attracting chemokines and spontaneously induce Th1, Th17 and Th2 cell activity. Arthritis Res Ther. (2013) 15:R155. doi: 10.1186/ar4338, PMID: 24286358 PMC3979121

[87] Miller JC Brown BD Shay T Gautier EL Jojic V Cohain A. Deciphering the transcriptional network of the dendritic cell lineage. Nat Immunol. (2012) 13:888–99. doi: 10.1038/ni.2370, PMID: 22797772 PMC3985403

[88] Tang KT Chen HH Chen TT Bracci NR Lin CC. Dendritic cells and antiphospholipid syndrome: an updated systematic review. Life (Basel). (2021) 11:801. doi: 10.3390/life11080801, PMID: 34440545 PMC8400181

[89] O’Keeffe M Mok WH Radford KJ. Human dendritic cell subsets and function in health and disease. Cell Mol Life Sci. (2015) 72:4309–25. doi: 10.1007/s00018-015-2005-0, PMID: 26243730 PMC11113503

[90] Halim TY Hwang YY Scanlon ST Zaghouani H Garbi N Fallon PG. Group 2 innate lymphoid cells license dendritic cells to potentiate memory TH2 cell responses. Nat Immunol. (2016) 17:57–64. doi: 10.1038/ni.3294, PMID: 26523868 PMC4685755

[91] Worbs T Hammerschmidt SI Förster R. Dendritic cell migration in health and disease. Nat Rev Immunol. (2017) 17:30–48. doi: 10.1038/nri.2016.116, PMID: 27890914

[92] Maruotti N Crivellato E Cantatore FP Vacca A Ribatti D. Mast cells in rheumatoid arthritis. Clin Rheumatol. (2007) 26:1–4. doi: 10.1007/s10067-006-0305-3, PMID: 16741781

[93] Rivellese F Rossi FW Galdiero MR Pitzalis C de Paulis A. Mast cells in early rheumatoid arthritis. Int J Mol Sci. (2019) 20:2040. doi: 10.3390/ijms20082040, PMID: 31027208 PMC6515166

[94] McNeil HP Gotis-Graham I. Human mast cell subsets–distinct functions in inflammation? Inflammation Res. (2000) 49:3–7. doi: 10.1007/PL00012386, PMID: 10778914

[95] Suurmond J Rivellese F Dorjée AL Bakker AM Rombouts YJ Rispens T. Toll-like receptor triggering augments activation of human mast cells by anti-citrullinated protein antibodies. Ann Rheum Dis. (2015) 74:1915–23. doi: 10.1136/annrheumdis-2014-205562, PMID: 24818634

[96] Rivellese F Suurmond J Habets K Dorjée AL Ramamoorthi N Townsend MJ. Ability of interleukin-33- and immune complex-triggered activation of human mast cells to down-regulate monocyte-mediated immune responses. Arthritis Rheumatol. (2015) 67:2343–53. doi: 10.1002/art.39192, PMID: 25989191

[97] Zhu J Paul WE. Heterogeneity and plasticity of T helper cells. Cell Res. (2010) 20:4–12. doi: 10.1038/cr.2009.138, PMID: 20010916 PMC3494736

[98] Ding Y-W Zhang Z-W Cui X-Y Hu R-Y Li Y Huang S-D. ZnCur nanoparticle-enhanced multifunctional hydrogel platform: Synergistic antibacterial and immunoregulatory effects for infected diabetic wound healing. Chem Eng. J. (2025) 503:158387. doi: 10.1016/j.cej.2024.158387

[99] Kim KW Kim BM Won JY Min HK Lee KA Lee SH. Regulation of osteoclastogenesis by mast cell in rheumatoid arthritis. Arthritis Res Ther. (2021) 23:124. doi: 10.1186/s13075-021-02491-1, PMID: 33882986 PMC8059019

[100] Shao C Fu B Ji N Pan S Zhao X Zhang Z. Alisol B 23-acetate inhibits igE/ag-mediated mast cell activation and allergic reaction. Int J Mol Sci. (2018) 19:4092. doi: 10.3390/ijms19124092, PMID: 30567287 PMC6320761

[101] Zhou Y Han D Follansbee T Wu X Yu S Wang B. Transient receptor potential ankyrin 1 (TRPA1) positively regulates imiquimod-induced, psoriasiform dermal inflammation in mice. J Cell Mol Med. (2019) 23:4819–28. doi: 10.1111/jcmm.14392, PMID: 31111624 PMC6584593

[102] Dominical VM Bértolo MB Almeida CB Garrido VT Miguel LI Costa FF. Neutrophils of rheumatoid arthritis patients on anti-TNF-α therapy and in disease remission present reduced adhesive functions in association with decreased circulating neutrophil-attractant chemokine levels. Scand J Immunol. (2011) 73:309–18. doi: 10.1111/j.1365-3083.2011.02503, PMID: 21208248

[103] Zhang W Chen Y Liu Q Zhou M Wang K Wang Y. Emerging nanotherapeutics alleviating rheumatoid arthritis by readjusting the seeds and soils. J Control Release. (2022) 345:851–79. doi: 10.1016/j.jconrel.2022.04.001, PMID: 35398172

[104] Xiang Y Jiang Y Lu L. Low-dose trypsin accelerates wound healing via protease-activated receptor 2. ACS Pharmacol Transl Sci. (2023) 7:274–84. doi: 10.1021/acsptsci.3c00263, PMID: 38230283 PMC10789143

[105] Chiappetta N Gruber B. The role of mast cells in osteoporosis. Semin Arthritis Rheumatol. (2006) 36:32–6. doi: 10.1016/j.semarthrit.2006.03.004, PMID: 16887466

[106] Kamathewatta KI Condello AK Kulappu Arachchige SN Young ND Shil PK Noormohammadi AH. Characterisation of the tracheal transcriptional response of chickens to chronic infection with Mycoplasma synoviae. Vet Microbiol. (2024) 294:110119. doi: 10.1016/j.vetmic.2024.110119, PMID: 38772075

[107] Vliagoftis H Befus AD. Rapidly changing perspectives about mast cells at mucosal surfaces. Immunol Rev. (2005) 206:190–203. doi: 10.1111/j.0105-2896.2005.00279, PMID: 16048550

[108] Zoltowska AM Lei Y Fuchs B Rask C Adner M Nilsson GP. The interleukin-33 receptor ST2 is important for the development of peripheral airway hyperresponsiveness and inflammation in a house dust mite mouse model of asthma. Clin Exp Allergy. (2016) 46:479–90. doi: 10.1111/cea.12683, PMID: 26609909

[109] Lin SJ Kuo ML Hsiao HS Lee PT Lee WI Chen JY. Cytotoxic function and cytokine production of natural killer cells and natural killer T-like cells in systemic lupus erythematosis regulation with interleukin-15. Mediators Inflamm. (2019) 2019:4236562. doi: 10.1155/2019/4236562, PMID: 31049024 PMC6462338

[110] Ding YW Li Y Zhang ZW Dao JW Wei DX. Hydrogel forming microneedles loaded with VEGF and Ritlecitinib/polyhydroxyalkanoates nanoparticles for mini-invasive androgenetic alopecia treatment. Bioact Mater. (2024) 38:95–108. doi: 10.1016/j.bioactmat.2024.04.020, PMID: 38699241 PMC11061199

[111] Ahern DJ Brennan FM. The role of Natural Killer cells in the pathogenesis of rheumatoid arthritis: major contributors or essential homeostatic modulators? Immunol Lett. (2011) 136:115–21. doi: 10.1016/j.imlet.2010.11.001, PMID: 21073898

[112] Edilova MI Akram A Abdul-Sater AA. Innate immunity drives pathogenesis of rheumatoid arthritis. BioMed J. (2021) 44:172–82. doi: 10.1016/j.bj.2020.06.010, PMID: 32798211 PMC8178572

[113] Wubben R Efstathiou C Stevenson NJ. The interplay between the immune system and viruses. Vitam Horm. (2021) 117:1–15. doi: 10.1016/bs.vh.2021.06.011, PMID: 34420576

[114] Zhao Y Lv J Zhang H Xie J Dai H Zhang X. Gene expression profiles analyzed using integrating RNA sequencing, and microarray reveals increased inflammatory response, proliferation, and osteoclastogenesis in pigmented villonodular synovitis. Front Immunol. (2021) 12:665442. doi: 10.3389/fimmu.2021.665442, PMID: 34248943 PMC8264543

[115] Zhu J Jia E Zhou Y Xu J Feng Z Wang H. Interleukin-22 secreted by NKp44+ Natural killer cells promotes proliferation of fibroblast-like synoviocytes in rheumatoid arthritis. Med (Baltimore). (2015) 94:e2137. doi: 10.1097/MD.0000000000002137, PMID: 26717357 PMC5291598

[116] Veenbergen S Bennink MB Affandi AJ Bessis N Biton J Arntz OJ. A pivotal role for antigen-presenting cells overexpressing SOCS3 in controlling invariant NKT cell responses during collagen-induced arthritis. Ann Rheum Dis. (2011) 70:2167–75. doi: 10.1136/ard.2011.154815, PMID: 21873688

[117] Veenbergen S Smeets RL Bennink MB Arntz OJ Joosten LA van den Berg WB. The natural soluble form of IL-18 receptor beta exacerbates collagen-induced arthritis via modulation of T-cell immune responses. Ann Rheum Dis. (2010) 69:276–83. doi: 10.1136/ard.2008.100867, PMID: 19188194

[118] Żyżyńska-Granica B Trzaskowski B Dutkiewicz M Zegrocka-Stendel O Machcińska M Bocian K. The anti-inflammatory potential of cefazolin as common gamma chain cytokine inhibitor. Sci Rep. (2020) 10:2886. doi: 10.1038/s41598-020-59798-3, PMID: 32076052 PMC7031511

[119] Yu CY Lee HS Joo YB Cho SK Choi CB Sung YK. Transcriptomic network analysis reveals key drivers of response to anti-TNF biologics in patients with rheumatoid arthritis. Rheumatol (Oxford). (2024) 63:1422–31. doi: 10.1093/rheumatology/kead403, PMID: 37572297

[120] Zhao S Grieshaber-Bouyer R Rao DA Kolb P Chen H Andreeva I. Effect of JAK inhibition on the induction of proinflammatory HLA-DR+CD90+ Rheumatoid arthritis synovial fibroblasts by interferon-γ. Arthritis Rheumatol. (2022) 74:441–52. doi: 10.1002/art.41958, PMID: 34435471 PMC9060076

[121] Sato K Takayanagi H. Osteoclasts, rheumatoid arthritis, and osteoimmunology. Curr Opin Rheumatol. (2006) 18:419–26. doi: 10.1097/01.bor.0000231912.24740.a5, PMID: 16763464

[122] Zhang C Tian Z. NK cell subsets in autoimmune diseases. J Autoimmun. (2017) 83:22–30. doi: 10.1016/j.jaut.2017.02.005, PMID: 28285736

[123] Zhang AL Colmenero P Purath U Teixeira de Matos C Hueber W Klareskog L. Natural killer cells trigger differentiation of monocytes into dendritic cells. Blood. (2007) 110:2484–93. doi: 10.1182/blood-2007-02-076364, PMID: 17626840 PMC1988958

[124] Lu Z Tian Y Bai Z Liu J Zhang Y Qi J. Increased oxidative stress contributes to impaired peripheral CD56dimCD57+ NK cells from patients with systemic lupus erythematosus. Arthritis Res Ther. (2022) 24:48. doi: 10.1186/s13075-022-02731-y, PMID: 35172900 PMC8848960

[125] Sokolov D Gorshkova A Tyshchuk E Grebenkina P Zementova M Kogan I. Large extracellular vesicles derived from natural killer cells affect the functions of monocytes. Int J Mol Sci. (2024) 25:9478. doi: 10.3390/ijms25179478, PMID: 39273424 PMC11395174

[126] Tong L Thumboo J Tan YK Wong TY Albani S. The eye: a window of opportunity in rheumatoid arthritis? Nat Rev Rheumatol. (2014) 10:552–60. doi: 10.1038/nrrheum, PMID: 24914693

[127] De Cauwer A Mariotte A Sibilia J Bahram S Georgel P. DICER1: A key player in rheumatoid arthritis, at the crossroads of cellular stress, innate immunity, and chronic inflammation in aging. Front Immunol. (2018) 9:1647. doi: 10.3389/fimmu.2018.01647, PMID: 30087677 PMC6066587

[128] Gulan G Ravlic-Gulan J Strbo N Sotosek V Nemec B Matovinovic D. Systemic and local expression of perforin in lymphocyte subsets in acute and chronic rheumatoid arthritis. J Rheumatol. (2003) 30:660–70., PMID: 12672182

[129] Zwirner NW Domaica CI. Cytokine regulation of natural killer cell effector functions. Biofactors. (2010) 36:274–88. doi: 10.1002/biof.107, PMID: 20623510

[130] Yamin R Berhani O Peleg H Aamar S Stein N Gamliel M. High percentages and activity of synovial fluid NK cells present in patients with advanced stage active Rheumatoid Arthritis. Sci Rep. (2019) 9:1351. doi: 10.1038/s41598-018-37448-z, PMID: 30718650 PMC6361912

[131] Vivier E Rebuffet L Narni-Mancinelli E Cornen S Igarashi RY Fantin VR. Natural killer cell therapies. Nature. (2024) 626(8000):727–36. doi: 10.1038/s41586-023-06945-1, PMID: 38383621

[132] Wagner JA Fehniger TA. Human adaptive natural killer cells: beyond NKG2C. Trends Immunol. (2016) 37:351–3. doi: 10.1016/j.it.2016.05.001, PMID: 27179621 PMC4885776

[133] Culemann S Grüneboom A Nicolás-Ávila JÁ Weidner D Lämmle KF Rothe T. Locally renewing resident synovial macrophages provide a protective barrier for the joint. Nature. (2019) 572:670–5. doi: 10.1038/s41586-019-1471-1, PMID: 31391580 PMC6805223

[134] Gossec L Baraliakos X McInnes I Kerschbaumer A de Wit M Dougados M. Response to: ‘Comment on: ‘EULAR recommendations for the management of psoriatic arthritis with pharmacological therapies: 2019 update’ by Gossec et al’ by Wei et al. Ann Rheum Dis. (2022) 81:e139. doi: 10.1136/annrheumdis-2020-218456, PMID: 32873555

[135] Blom AM Nandakumar KS Holmdahl R. C4b-binding protein (C4BP) inhibits development of experimental arthritis in mice. Ann Rheum Dis. (2009) 68:136–42. doi: 10.1136/ard.2007.085753, PMID: 18276745

[136] Li Y Woods K Parry-Strong A Anderson RJ Capistrano C Gestin A. Distinct dysfunctional states of circulating innate-like T cells in metabolic disease. Front Immunol. (2020) 11:448. doi: 10.3389/fimmu.2020.00448, PMID: 32231670 PMC7082397

